# Paediatric, pedestrian road traffic injuries in the city of Mashhad in north-eastern Iran 2015–2019: a data note

**DOI:** 10.1186/s13104-020-05203-1

**Published:** 2020-07-31

**Authors:** Parinaz Tabari, Hamidreza Shabanikiya, Nasser Bagheri, Robert Bergquist, Soheil Hashtarkhani, Fatemeh Kiani, Alireza Mohammadi, Behzad Kiani

**Affiliations:** 1 School of Allied Medical Sciences, Tehran University of Medical Sciences, Tehran, Iran; 2 Clinical Education Research Center, Shiraz University of Medical Sciences, Shiraz, Iran; 3 Social Determinants of Health Research Centre, Mashhad University of Medical Sciences, Mashhad, Iran; 4 Visualization and Decision Analytics (VIDEA) Lab, Centre for Mental Health Research, Research School of Population Health, College of Health and Medicine, The Australian National University, Canberra, Australia; 5 Ingerod, Brastad, Sweden; 6 Department of Medical Informatics, School of Medicine, Mashhad University of Medical Sciences, Mashhad, Iran; 7 Department of Geography & Planning, Faculty of Humanities, University of Mohaghegh Ardabili, Ardabil, Iran

**Keywords:** Paediatric Accident, Pedestrian Accident, Road Traffic Injuries, Iran, Mashhad, Spatial data, Non-spatial data, Geographic Information Systems

## Abstract

**Objectives:**

The leading factors of paediatric, pedestrian road traffic injuries (PPRTIs) are associated with the characteristics of immediate environment. Spatial analysis of data related to PPRTIs could provide useful knowledge for public health specialists to prevent and decrease the number of accidents. Therefore, we aim to release the datasets which have been used to conduct a multiple-scale spatial analysis of PPRTIs in the city of Mashhad, Iran, between 2015-2019.

**Data description:**

The data include four datasets. The base PPRTIs dataset includes motor vehicle accidents and their attributes in the city of Mashhad between March 2015 and March 2019. The attribute data includes the month, day of the week, hour of the day, place (longitude and latitude) of each accident, age range of the child and gender. Furthermore, three spatial datasets about the city of Mashhad are introduced; (1) the digital boundaries of Neighbourhood, (2) road network dataset (street lines) and (3) urban suburbs of Mashhad.

## Objective

Pedestrians are implicated in up to 22% of all traffic deaths worldwide [1]. According to the report on road traffic injuries that was released by the World Health Organization (WHO), the leading factors for pedestrian accidents are associated with the characteristics of immediate environment [2]. An essential course of actions in designing any policy planning and interventions to reduce the number of accidents is to determine high-risk areas where environmental conditions may play a role. Although the number of deaths caused by road traffic accidents in Iran declined between the years 2015 and 2018, the rate of traffic accident related mortality is still considerably high [3]. Khorasan-Razavi has the second-highest number of road traffic accidents in Iran and 76% of all road casualties in Khorasan-Razavi occur in Mashhad, which is the capital city of this province [4]. The majority of underprivileged children in Mashhad live in suburban areas with low socioeconomics status, and they experience more psychological distress and physical conditions, and this may increase their susceptibility to accidents. Therefore, paediatric, pedestrian road traffic injuries (PPRTIs) should be a priority for policy-makers to develop tailored strategies to reduce accident-related injuries for children.

Environmental and geographical considerations of the spatial trends of people’s behavior should be taken into account. In this context, we employed a multiple scale spatial analysis and identified high-risk neighbourhoods of the city of Mashhad with particular reference to high-risk streets [5]. The mentioned analysis is briefly described in the next section. The extensive datasets with respect to PPRTIs in Mashhad, its communities and streets, between 2015 and 2019 are offered to provide the details of the spatial data collected and their use in future studies of traffic accidents involving children in Mashhad and elsewhere.

## Data description

Geographic Information System (GIS) is a tool that supports spatial analysis and, more specifically, point pattern analysis [6]. GIS data have two dimensions; spatial and non-spatial [7]. The former component refers to the location or geographical shape of an entity, while the latter describes the entity itself (attributes and temporal data) [8]. For example, a PPRTI has an address that shows the location of the accident (spatial component) and attributes such as age group, gender, date/time of the accident (non-spatial components). We randomly jittered the latitude and longitude of the accidents into a 100-m buffer to avoid potential identification of children. Furthermore, the age data were grouped into four categories: up to 3, between 3 and 5, between 5 and 12, and between 12 and 19.

Emergency care calls in the city of Mashhad from March 2015 to March 2019 were extracted from the Emergency Medical Services (EMS) database and the PPRTIs were developed through data processing. The EMS database contains textual explanations of emergency missions performed by the ambulance services. All records of the stated database were then investigated to obtain the PPRTI-related records (Data set 1), which includes latitude and longitude of the accident, month, day of the week and hour of the day as well as the age group and gender of the child involved. Having been recorded in the Persian language, the addresses had to be geocoded manually using the software Google MyMaps (http://www.google.com/mymaps). These data were subsequently transformed into a Keyhole Markup Language (KML) file and imported to ArcGIS software version 10.6 (ESRI, Redands, CA, USA) for further spatial analysis. The file was a point-density map and the gender-based geographic distribution of PPRTIs were subsequently extracted. The local PPRTI hotspots based on children’s age and gender were then investigated using a geographical grid network at the neighbourhood level by applying Getis-Ord Gi* method and the Anselin Local Moran’s *I* statistic. This approach assisted in obtaining high-age and low-age PPRTI clusters by utilizing the spatial data of Mashhad neighbourhoods and the city area (Data files 1, 2). Two clusters (High–High and Low–Low) and two outliers (High-Low and Low–High) of PPRTIs were also determined by employing the Anselin Local Moran’s *I* statistic. The city’s street vector layer was offered by the municipality of Mashhad (Data file 3). Through the buffer analysis method, all streets were classified based on the PPRTI rate. In addition to the creation of the streets’ 10-m buffer, the number of PPRTIs for each street was also extracted by the spatial join methodology. Streets were then classified into four grades: low-risk (1st), middle-low risk (2nd), middle-high risk (3rd) and high-risk streets (4th). Table 1 shows the details of each dataset and provides links to access them.

**Table 1:** Overview of data sets

Dataset (label)	Name of data file/data set	File types (file extension)	Data repository and identifier (DOI or accession number)
Data set 1	PPRTIs-1	MS Excel file (.xlsx)	Harvard Dataverse (10.7910/DVN/9XVUQX) [9]
Data file 1	Mashhad neighbourhoods	Shape file (.shp)	Harvard Dataverse (10.7910/DVN/9XVUQX) [9]
Data file 2	Mashhad border	Shape file (.shp)	Harvard Dataverse (10.7910/DVN/9XVUQX) [9]
Data file 3	Mashhad streets	Shape file (.shp)	Harvard Dataverse (10.7910/DVN/9XVUQX) [9]

## Limitations

People who evacuated the site of accidents without contacting the EMS, as well as those who were driven to hospitals by personal cars, have not been registered. This constraint understates the findings to some (unknown) degree. However, the identification of high-risk and low-risk areas should not be affected by this limitation. For subsequent surveys, it is recommended that hospitals and EMS data be integrated and linked in order to acquire information about all types of emergency cases.

## Data Availability

The data described in this Data note can be freely and openly accessed on the Harvard Dataverse under (10.7910/DVN/9XVUQX) [9]. Please see Table 1 and reference list for details and link to the data.

## Abbreviations

PPRTI: Paediatric, Pedestrian Road Traffic Injuries
WHO: World Health Organization
GIS: Geographic Information System
EMS: Emergency Medical Services
KML: Keyhole Markup Language
